# Intraspecific Phenotypic Variation and Morphological Divergence of Strains of *Folsomia candida* (Willem) (Collembola: Isotomidae), the "Standard" Test Springtaill

**DOI:** 10.1371/journal.pone.0136047

**Published:** 2015-09-10

**Authors:** Thomas Tully, Mikhail Potapov

**Affiliations:** 1 Institute of ecology and environmental sciences—Paris (iEES Paris, UMR 7618), CNRS, UPMC Univ Paris 06, Sorbonne Universités, Paris, France; 2 ESPE de Paris, Université Paris 4, Sorbonne Universités, Paris, France; 3 Department of Zoology and Ecology, Moscow State Pedagogical University, Moscow, Russia; Roehampton university, UNITED KINGDOM

## Abstract

We describe and compare the external morphology of eleven clonal strains and one sexual lineage of the globally distributed *Folsomia candida*, known as “standard” test Collembola. Of the 18 morphological characters studied, we measured 14 to have significant between-strains genetic variations, 9 of these had high heritabilities (>78%). The quantified morphological polymorphism was used to analyse the within-species relationships between strains by using both a parsimony analysis and a distance tree. These two detailed morphological phylogenies have revealed that the parthenogenetic strains grouped themselves into two major clades. However the exact position of the sexual strain remains unclear and further analysis is needed to confirm its exact relationship with the parthenogenetic ones. The two morphologically based clades were found to be the same as the ones previously described using molecular analysis. This shows that despite large within-strain variations, morphological characters can be used to differentiate some strains that have diverged within a single morphospecies. We discuss the potential evolutionary interpretations and consequences of these different levels of phenotypic variability.

## Introduction

Springtails belong to a very ancient group (Class Collembola, ~410 Mya) [1] which has colonised nearly all terrestrial habitats [2]. This taxon comprises about 8000 described species and is considered to be moderately diversified [2,3]. New species are described regularly, mostly on the basis of external morphology [4]. But the real number of existing species of Collembola on Earth is difficult to predict although a ballpark estimation of about 50,000 species has been proposed [5]. It seems also reasonable to assume that the current species' diversity is considerably underestimated, especially if one takes into account molecular studies that have recently revealed the existence of extensive cryptic genetic diversity in several widely distributed species or complexes of species [3,6–11].

To recognise, describe and define species in this group, taxonomists rely essentially on the chaetotaxy [4]. Using the chaetotaxy, they describe and compare the number, position and shape of external sensillar elements, searching for diagnostic characters even in minute species [11].

Infraspecific character variation is commonly observed in Collembola chaetotaxic studies [12–24] and for pragmatic reason it is normally considered as problematic rather than informative [25]. Some common setae vary considerably in number between individuals or even from one side to the other on the same individual. They also “float” making the determination more complicated [26]. Other setae are much more stable and are useful for interspecific identification [4,25,27].

The parthenogenesis is widely known in Collembola [28,29] and the morphological species concept which is used for practical purposes in the springtails is especially relevant for such a mode of reproduction for which the biological species concept can not be applied. In parthenogenetic species, the gene flow is disrupted between lineages. Each strain thus forms an independent evolutionary unit, as do different species that have recently diverged [30,31]. Different strains may have diverged owing to genetic drift of different selective pressures if they evolve in contrasted environments. Thus studying the infraspecific morphological character variation in a parthenogenetic species may be of interest for studying the early steps of morphological divergence during an on-going speciation event and understand species and their origins in asexual organisms [31].

Our work focuses on the evolution of infraspecific character variation in the parthenogenetic springtail *Folsomia candida*, one of the most commonly used laboratory Collembola [32]. This springtail is cosmopolitan [9,32] and its range spans every terrestrial ecozones. In Europe, it occupies mostly protected soils (flower pots, greenhouses, caves) though it can be collected in many open habitats being more frequent in more disturbed sites. This morphospecies is known to reproduce mainly asexually [32] although some populations are sexual [33].

Due to its worldwide distribution and to the ease with which it can be raised and maintained in the laboratory, this species became a standard micro-arthropod and is now used in many laboratories as a model organism in different domains of soil biology [32]. It is widely used especially as a model organism for ecotoxicological testing purposes [34–37].

Several strains from different geographical origins have been collected and used by various laboratories [9]. But most experiments are conducted on one [35,38–40] or a few clonal strains [41–43]. If the genetic homogeneity of different individuals belonging to a single strain is a clear benefit for standardisation in the laboratory experiments, it can also lead to a delusion as regards the relevance of comparisons that can be made between studies made on different strains. In other words, because of disclosed cryptic genetic diversity in *F*. *candida*, this Collembola may not be an ideal “standard” as it is supposed to be [44].

Indeed, some infraspecific genetic polymorphism has been described using allozyme techniques, RAPD markers or gene sequences [9,41,42]. In a previous study where 11 strains were compared, we found that this within-species genetic diversity is organised in two major lineages [9]. Although some genetic differences are found between different strains within each lineage, major and consistent differences have been observed between these two clades. It was later shown that two contrasted biodemographic strategies evolved along with the early divergence of these two major branches of the evolutionary tree. These two main strategies differ in their overall reproductive and survival potentials and on their level of adaptive plasticity [45,46]. One can easily see that making use of strains belonging to one branch or the other of this species evolutionary tree may strongly influence the outcome of any laboratory experiment.

Our aim here is to study within this commonly used morphospecies (*F*. *candida* sensu stricto, see below) whether external morphology can be used to make sense of the cryptic genetic diversity and less cryptic life history diversity, which have previously been described.

More precisely we aim to address the following four points:

- **The description of morphological variation.** How stable is the external morphology in this morphospecies? Can we see some morphological trait variation and if so what are the traits that are found to vary? Does this variation put in question the taxonomical status of this morphospecies?
- **The quantification of morphological variation.** By comparing the morphology of individuals belonging to different strains, we can describe the patterns of morphological variation within and amongst lineages of a single species. This will enable us to know whether the traits that vary between strains also vary between individuals within a lineage. In other words, it will enable us to partition the total morphological variances of traits into their additive genetic components (between-lineage variance) and their random environmental or developmental components. This is required to quantify the heritabilities of the morphological traits that are found to vary.
- **The evolutionary significance of morphological variation.** Estimating the heritabilities of traits is important since it permits one to estimate the level of morphological divergence between the strains and to know how far these strains are on the early stages of an ongoing speciation process. Moreover, the quantification and partitioning of the morphological variation can be used to build up a phylogeny of the different lineages, and to question whether this phylogeny is congruent with the ones derived from molecular analysis. This will show us if the morphology, which is supposed to carry some reliable information for taxonomy, is also relevant for disentangling the evolutionary relationships of different lineages within a single morphospecies.
- **The practical use of morphological variation.** The present work also aims at searching for characters that could be used to reliably identify some lineages of this commonly used species. This could enable laboratories to easily verify whether they are working on common clades or on different ones.

## Material and Methods

### Species' taxonomical description

The springtail *Folsomia candida* was first described in 1902 by Willem [47] from a single individual found in a Belgian cave. It was later morphologically characterised in greater detail [48]. Afterwards this species was repeatedly redescribed following the understanding of Stach (for details see [49]). So far it is not considered to be a good morphospecies–Potapov et Gao [50] proposed to use two terms *F*. *candida* sensu lato and *F*. *candida* sensu stricto. The former is more tentative as including all forms or species closely related to “typical” *F*. *candida* greatly differing in body size, number of chaetae on furca, foil-chaetae and other features. *F*. *candida* sensu lato is more known from Asia and America. *F*. *candida* s.str. was defined basing on European strains kept in the laboratory of the first author of the paper, its diagnosis is much narrower [50]. In the present study we deal with *F*. *candida* s.str.

### Lineages examined

Twelve laboratory strains of *F*. *candida* have been examined, and labelled AP, BR, BV, DK, GB, GM, HA, PB, SH, TO, US, WI. All of them except SH reproduce asexually and have been kept in the laboratory since 1999. Apart from the strain SH, all were previously studied according to their geographical origin and molecular phylogeny [9] and to some of their life-history traits [45,46]. As mentioned above, these previous studies have shown that these strains can be grouped into two major clades. The first clade (A) encompasses five lineages (AP, GB, HA, BR & BV) with a low reproductive potential but a relatively high longevity. The second clade (B) is composed of six lineages (DK, GM, PB, TO, US & WI) with a higher reproductive potential and a higher mean death rate [45,46]. As regards the strain SH, it comes from a population in Shanghai (China) and is composed of male and female individuals.

To exclude potential age-dependent morphological variability, only young adults were used for this study. They were all grown from eggs, received the same food and maintained in similar containers at the same temperature (21°C) to standardise conditions, which could influence the morphology. Note that SH was not cultivated in the same laboratory and we cannot exclude the fact that slightly different conditions could influence their morphology. All of the studied SH were females. For each strain, about twelve young adults having approximately the same size (~1.2/1.8 mm) were taken from these standard cultures and stored in alcohol.

A total of 66 individuals of *F*. *candida* with about 8 to 12 individuals per strain have been examined. More abundant material could give more characters to be used to separate the strains statistically.

Note that for each strain, the collected individuals were separated into two tubes and the 2*12 = 24 tubes were anonymised by the first author and then given to the second author for examination. The strain identity was later reattributed to the 24 groups of individuals after their character description was completed.

### Morphological characters

**Fig 1:**
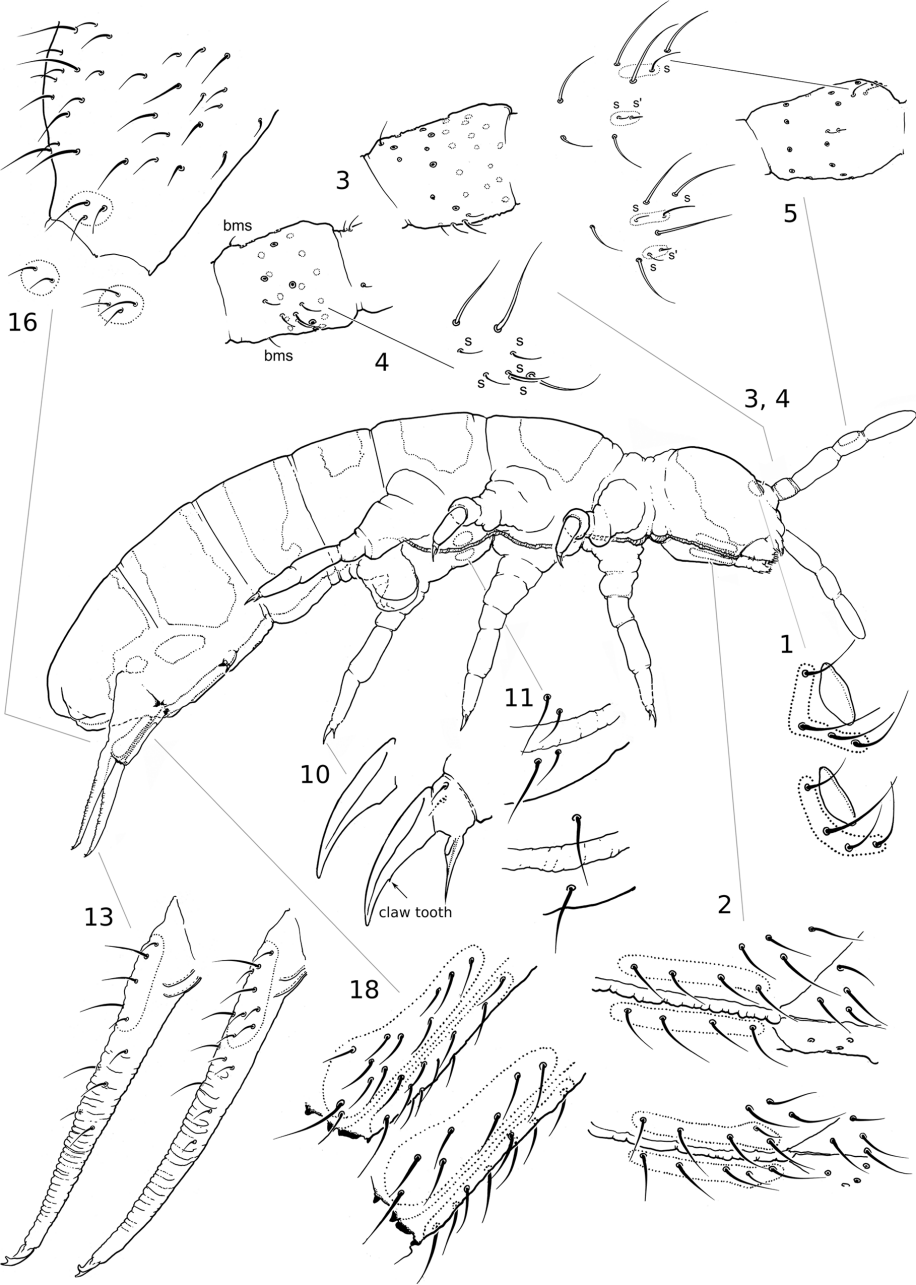
External morphology. External morphology of *Folsomia candida* displaying the main observed characters and their variation. The character numbers correspond to the one in Table 1. *bms*, basal microsensilla.

The springtails were mounted on microscope slides in Phoera liquid (200 g of chloral hydrate, 20 g of glycerol and 30 g of gum arabic dissolved and mixed into 50 g of distilled water) and their morphology was carefully studied. The specimens were first examined to verify that their chaetotaxy corresponded to the diagnostic characters of the morphospecies [49]. Then 18 external morphological characters easy to observe with a standard light microscope on individuals mounted on a slide were systematically examined. These characters are listed and described in Table 1 and the main discriminant characters are illustrated in Fig 1. Almost all of these characters have been somewhat used in species’ taxonomy of the genus *Folsomia* [27], but their taxonomical values are different and depend on group of species. Additional sensilla on dorsal side of Ant.III (not shown) was not used because of its high variability within a strain. In addition, it is often difficult to recognise and calculate seta-like sensilla on this side of Ant.III. Many other characters were left out of our consideration here because they predictably displayed no or very low variability between our strains since we dealt with one species. We tried to observe every character on each studied individual although all the characters were not always visible. Obvious aberrations were not taken into consideration.

**Table 1:** Characters description and level of variation. The whole range of character variation is given. Data in parenthesis represent variants occurring singly. The whole dataset is available in S1 Dataset.

Body part	(Character number), name and description.	Range of character variation (outliers); Threshold (/)	Type of model and overall differences between clones and	Estimation of the character heritability h^2^.
Head	(01) The second guard seta along posterior edge of Post-Antennal Organ (PAO). The second seta is in group of guard setae of PAO. It is located near the middle part of PAO and is normally longer than the others.	At a level with other guard setae / behind other guard setae	Binomial (1 for “at the level”), χ^2^_11_ = 133, P < .001	94%
	(02) Nb. of post-labial setae on one side. All strains bear at least 4 large basal post-labial setae on one side. The variant of 5 setae means that an additional smaller seta appears between the first and second basal setae.	4–5; 4 / 5	Binomial, χ^2^_11_ = 135, P < .001	78%
	(03) Nb. of setae on Ant.I. Counted on one side. Only common setae are counted, sensilla and basal microsensilla are out of consideration. Basal microsensilla are shorter and thinner than common setae and are located dorsally and ventrally on the basal part of the segment.	18–32; ≤ 24 / >24	Poisson, χ^2^_11_ = 27, P = .005	4.4%
	(04) Nb. of sensilla on Ant.I. Total numbers of sensilla are reported here. There is invariably one long sensilla associated with generally 3 (2 to 4) short sensilla. Most of the character variation being between 4 and 5, we considered to have only two states: 5 or less than 5.	4–5; 4 (rarely 5) / 5 in all individuals of strain.	Binomial, χ^2^_11_ = 79, P < .001	86%
	(05) Sensilla on lateral side of Ant.III. Nb. of sensilla (0, 1, or rarely 2) located just posterior to lateral outer sensillum of antennal organ of third antennal segment. On right and left sides (usually visible on one side only). The additional sensillum is located just posterior to lateral outer sensillum of antennal organ of third antennal segment. It is shorter and thinner than the latter one.	0–1 (2); absent in all individuals of strain / present in all or some individuals of strain	Binomial, χ^2^_11_ = 107, P < .001	94%
Thorax	(06) Nb. of setae on upper subcoxa of Leg II	3–4	Binomial, χ^2^_11_ = 19.4, P = .054	
	(07) Nb. of setae on lower subcoxa of Leg II	6–11	Poisson, χ^2^_11_ = 5.8, P = .88	
	(08) Nb. of setae on upper subcoxa of Leg III	4–9	Poisson, χ^2^_11_ = 5.6, P = .90	
	(09) Nb. of setae on lower subcoxa of Leg III	7–14	Poisson, χ^2^_11_ = 17, P = .08	
	(10) Tooth on claw. Inner tooth is visible in laterally positioned claw and is normally estimated on the third pair of leg. The state “present” means that the tooth is well visible; irregularity in curvature of inner edge of claw is considered the absence.	absent or indistinct / present	Binomial, χ^2^_11_ = 137, P < .001	88%
	(11) Nb. of ventral setae on Th.III. Counted on one side.	1–4; 1 on both or one side / 2 or more on both or one side	Binomial, χ^2^_11_ = 113, P < .001	78%
Abdomen	(12) Nb. of anterior setae on dens.	22–38	Poisson, χ^2^_11_ = 24.2, P < .001	6.7%
	(13) Nb. of posterior setae in basal group on dens. All setae on posterior side of dens can be divided to a basal and two proximal groups: outer and inner. The basal group is located on strictly posterior part of dens lacking crenulations. As a rule, the mentioned groups are separated by gaps; in some individuals they are less distinct.	4–9; ≤ 7 / ≥ 6	Poisson, χ^2^_11_ = 37, P < .001	12%
	(14) Nb. of posterior setae in outer-proximal group on dens.	2–4	Binomial, χ^2^_11_ = 88, P < .001	80%
	(15) Nb. of posterior setae in inner-proximal group on dens.	2–4	Binomialχ^2^_11_ = 20, P = .04	15%
	(16) Nb. of apical setae on posterior side of manubrium.	(0–1) 2–4 (5); ≤ 2 / ≥ 2	Binomialχ^2^_11_ = 57, P < .001	94%
	(17) Nb. of posterior (dorsal) setae in lateral parts of manubrium (right/left).	(0) 1–2 (3)	Binomial, χ^2^_11_ = 122, P < .001	85%
	(18) Nb. of anterior setae on manubrium. Measured on both sides. One or two setae on lateral sides of manubrium are not included to the group of anterior setae.	11–27; ≤ 18 / ≥ 18	Poisson, χ^2^_11_ = 59, P < .001	12%

### Statistical analysis

#### Character analysis

**Fig 2:**
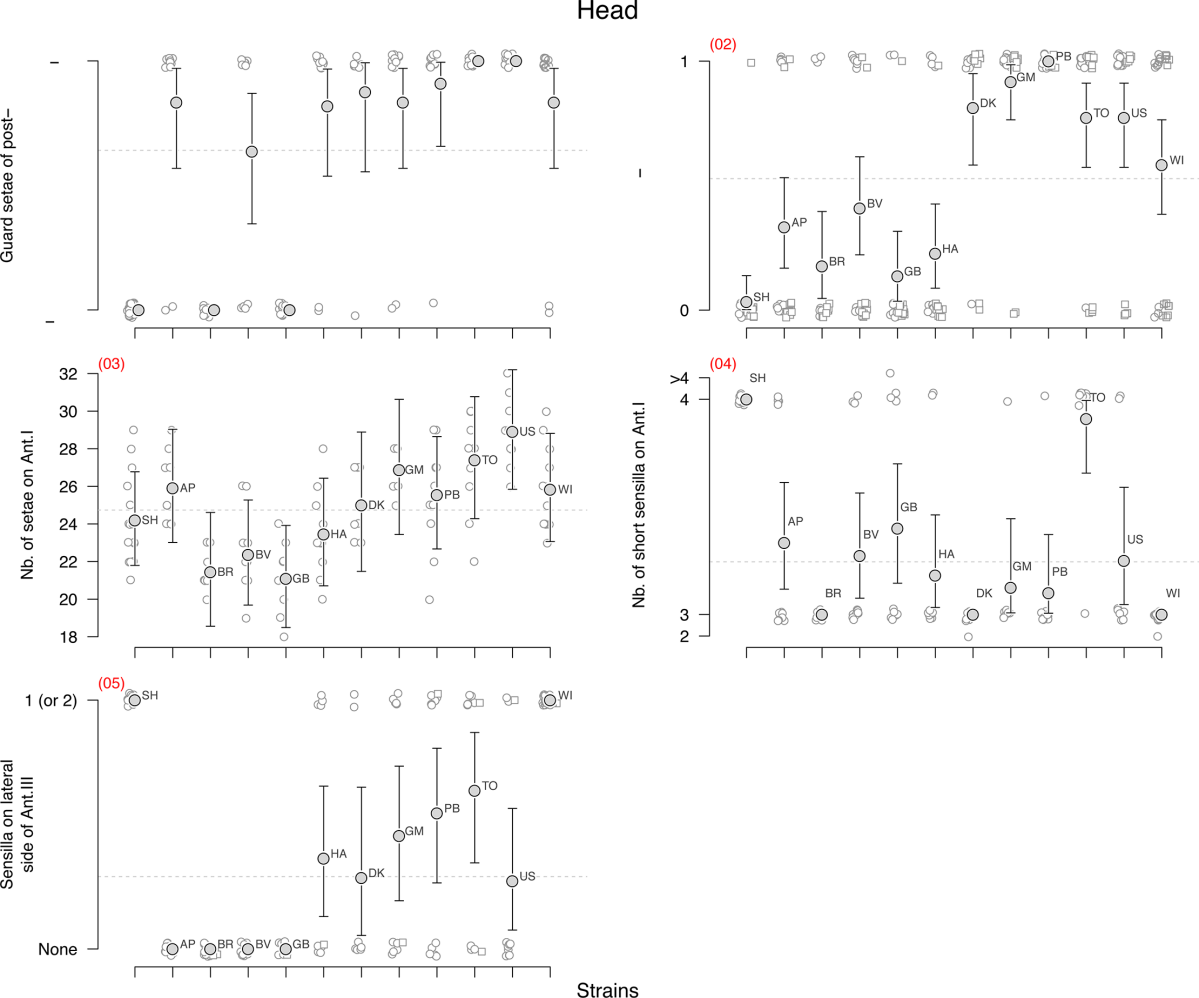
Head. Head morphological character state distribution (Table 1) for the studied strains of *F*. *candida*. The strains are aligned as follows: the new SH sexual strain is plotted on the left hand side. Then, based on previous work, we have grouped together the clones AP to HA since they are known to be genetically affiliated. The right hand side of the graphs represents in alphabetic order the clones DK to WI that are known to belong to another distinct clade. For each strain the raw observations are plotted with little vibration so that the points do not overlap. For lateral characters, the left side characters are figured as circles and the right side ones as squares. The estimated means and 95% confidence intervals (see main text for details, especially for characters treated as binomial variables) are plotted for each strain. The horizontal grey dotted line underlines the overall mean trait value.

**Fig 3:**
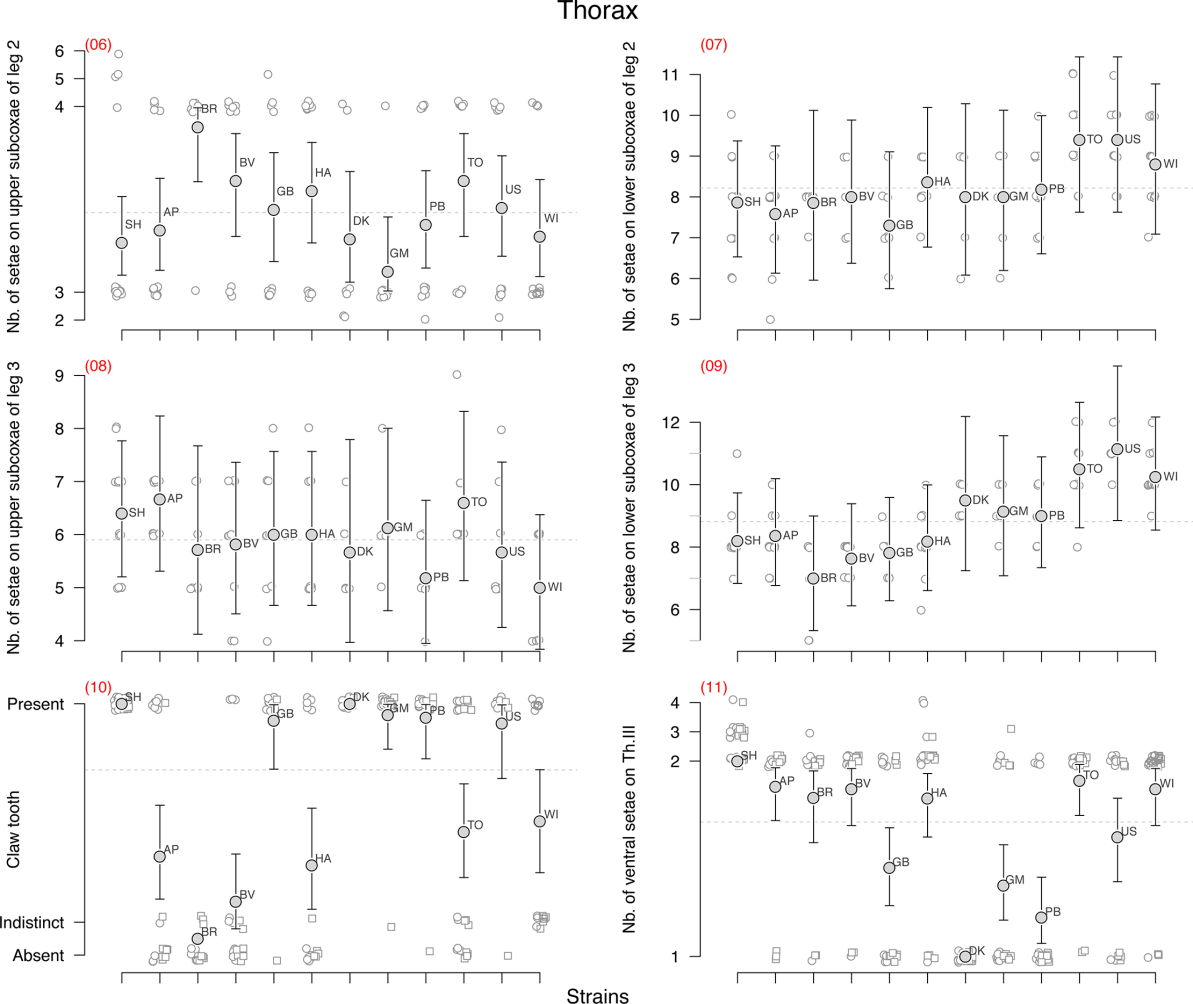
Thorax. Thorax morphological character state distribution for the studied strains of *F*. *candida*. See Fig 2 caption and Table 1 for details.

**Fig 4:**
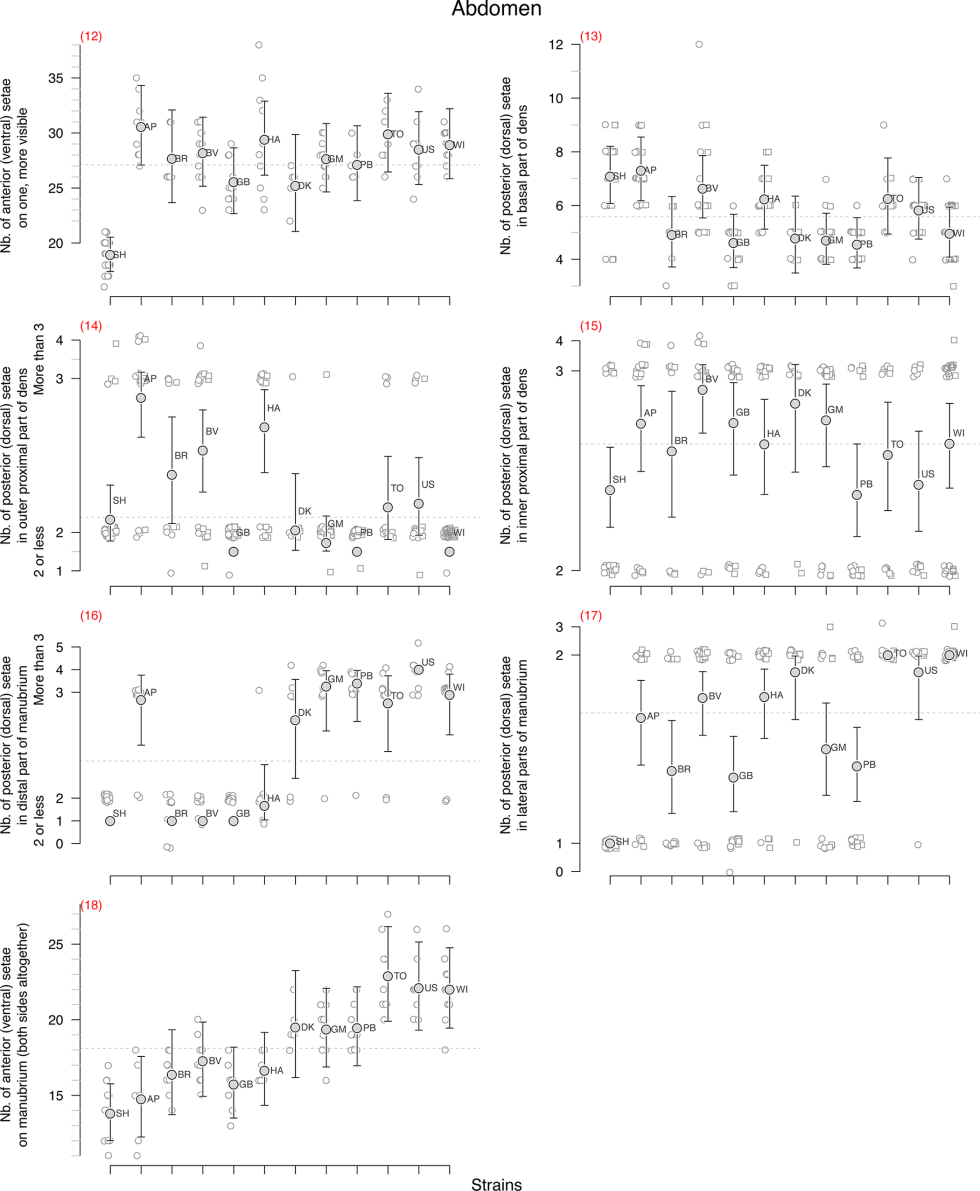
Abdomen. Abdomen morphological character state distribution for the studied strains of *F*. *candida*. See Fig 2 caption and Table 1 for details.

We analysed the variability of each character using either a binomial linear model for dichotomous characters such as the presence of an additional post-labial setae (character n°2 in Table 1) or a Poisson model for count characters with large number of values such as the number of setae on Ant.I (n°3) which varies for instance between 18 and 32. But most count characters displayed an almost binomial distribution. By that we mean that for these characters, most of the observed variation spreads between two values with some rare extra-values. For instance, about 95% of the observed springtails had either 3 or 4 setae on upper subcoxae of leg 2 (n°6) whereas only 4% had two and one individual had five setae. For this kind of character, we grouped together the outliers and rare values with their nearest most common ones in order to analyse the data as a binomial variable. In the former case, 2 and 3 are grouped together and 4 and 5 form the second group. This was done for characters n°4, 5, 6, 10, 11, 14, 15, 16 and 17. But in order to show the range of variation of the observed characters we have conserved the different observed possible values in the graphs (Figs 2, 3 and 4). The outliers in these graphs are plotted close to their nearest more dominant values so that the predicted mean and confidence interval derived from the binomial model could be plotted on top of the raw data (Figs 2, 3 and 4).

Binomial or Poisson linear models were used to search and test for any potential differences between strains. We used the *glm* (generalised linear model) function from the R statistical package to fit these models [51]. For each character, an appropriate *glm* model was fitted to the data with strain labels as covariate to test whether the trait differs globally between the strains (p values in Table 1). This analysis was used to sort out the characters that displayed significant differences between strains from those with no overall differences.

Similar *glm* models with strain labels as covariate but without the intercept were used to derive the predicted means and 95% confidence intervals for each character and strain (Figs 2, 3 and 4, Table 1). Only the characters that displayed some significant genetic variation were later used to measure their heritability and to build up a distance cladogram and a phylogenetic tree.

#### Characters’ heritabilities

We estimated the broad sense heritabilities of the characters that were found to differ significantly between strains. For clonal organisms, the relevant measure of genetic variance is the broad-sense heritability defined as the ratio of the among-clone component of variance to the total phenotypic variance: H^2^ = σ^2^_G_/σ^2^_T_ where σ^2^_G_ is the genetic variance (between strain variance) and σ^2^_T_ is the total trait variance [52]. We used the *glmmPQL* function from the *MASS* package to do such estimations. *glmmPQL* fits a generalised linear mixed model using penalised quasi-likelihood. In our case this model has been fitted to binomial or Poisson data depending on the characters.

We used no fixed effect but introduced in the model the strain label variable as a random factor to partition the overall character variance between “between-strain” variance and “within-strain” variance which enables the model to estimate the proportion of total character variation due to differences between strains, thus providing a surrogate of the broad sense heritability of the characters [52,53] (Table 1).

#### Phylogenetic analysis

**Fig 5:**
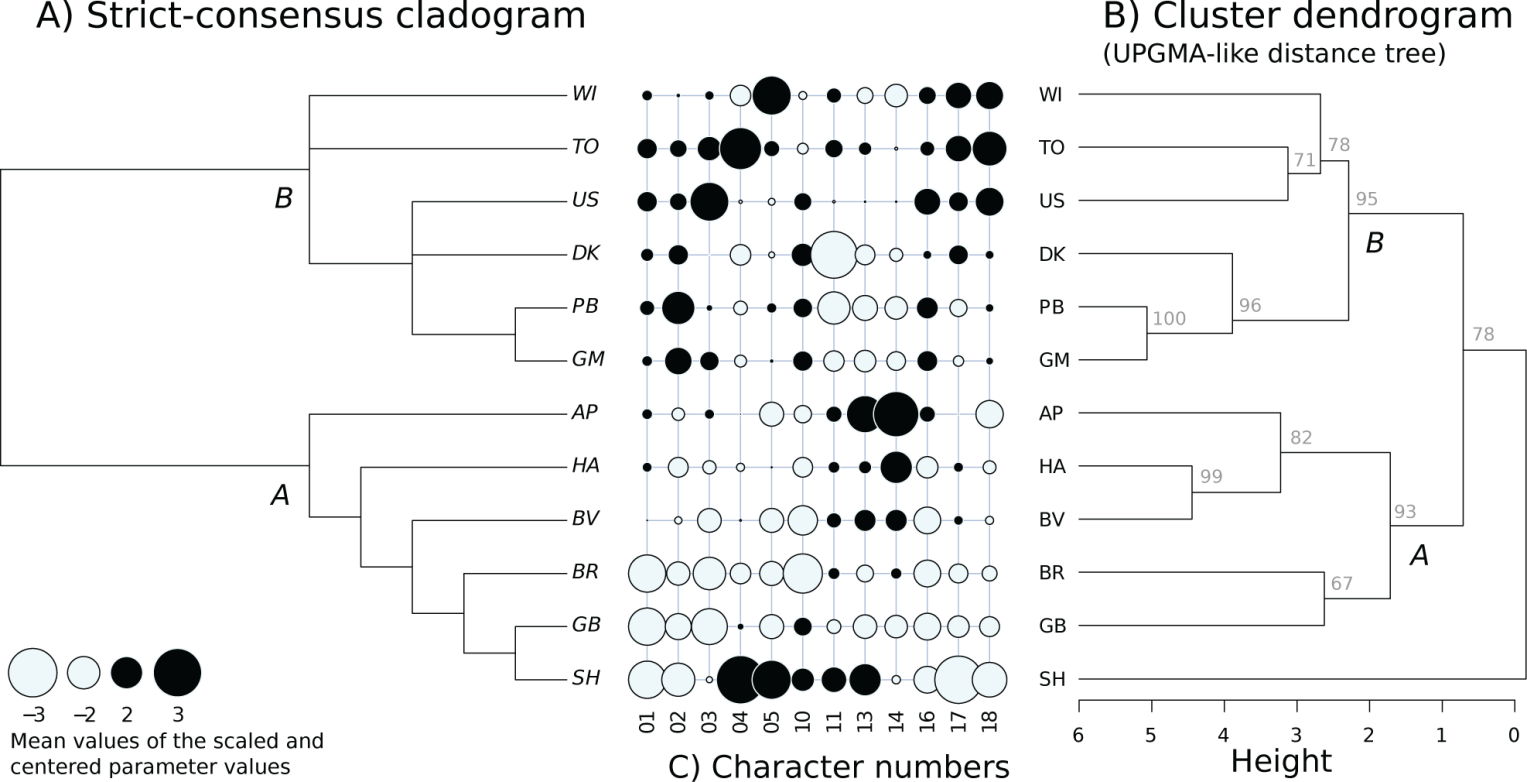
Phylogeny. A) Strict consensus tree obtained from a parsimony analysis of tree based on the binomilaisation of the scaled and centred morphological data (black and white colours of the trait values in C). B) Cluster dendrogram made on the scaled measurements of the morphological traits that have been shown to vary between strains using average method. The approximately unbiased (au) p-values (expressed as %) computed by multiscale bootstrap resampling indicate for each node how strong the cluster is supported by data.

We first studied the relationship between the strains using a cladistic approach. The character coding in the matrix of character was done as follows. We scaled and centred the matrix of the estimated mean per strain values of the morphological variables and transformed this matrix into a binomial matrix using the sign of each transformed measurement. For each character, the strains whose means are lower than the average mean have a state “0” whereas the other ones have a state “1” (black and white colours in Fig 5C). Here, we only used the morphological characters that were found to significantly vary between the strains. This matrix was transformed into an object of class *phyDat* (*phangorn* library in R) and then processed by the *bab* function (*phangorn* library) which finds all the most parsimonious trees after examining all possible trees [54,55]. We then used the *consensus* (*ape* library) function in order to obtain the strict consensus tree from the most parsimonious trees. This consensus tree was then artificially rooted between the two previously described clades (clades labelled A and B in Fig 5A) for graphical homogeneity [9].

#### Cluster analysis

The cladistic cladogram was complemented with a phenetic distance tree dendrogram. To build-up this dendrogram from our morphological data, we used a hierarchical cluster analysis (*hclust* function in program R 2.15) with an average linkage method which is the one used for UPGMA (Unweighted Pair Group Method of Aggregation). This was performed on the matrix of the scaled (centred and standardised) mean values for each strain of the characters that were shown to vary significantly (Fig 5C). Multiscale bootstrap resampling (*pvclust* function from the *pvclust* library) was used to provide approximately unbiased p-values (expressed as %) for each node, which indicate how strong the clusters are supported by the data. This enabled to highlight the clusters that are highly supported by the data [56] (Fig 5B).

## Results

### Characters’ variability and heritability

All individuals could be identified as the previously recognized *F*. *candida* morphospecies sensu strictu [50].

Some phenotypic variation was observed for all 18 characters under study but the level of genetic variation varied a lot between characters.

No genetic variation was found for four characters: the number of setae on all subcoxa of second and third legs (n° 6–9, Fig 3) strongly varied depending on the specimen but this variation was not found to be structured between the strains.

Some significant genetic variation associated with low heritability (h^2^<15%) was found for five characters (n° 3, 12, 13, 15, 18, Table 1), four of which were counts of setae (Poisson distributed data). These low heritabilities seemed to be due to the large within-strain character variation with large overlapping of the character distribution between the clones. For the number of anterior setae on dens (n°12), all the clonal strains were found to be very similar, the only observed genetic variation coming from the sexual strain SH which had on average less setae than the other strains. Aberrant asymmetry of dens often occurs–many individuals bear a different number of setae on the right and left dens (24 and 30, 27 and 35, as examples). Some differences between strains could probably be found if more specimens were studied.

Nine characters have high heritabilities (n° 1, 2, 4, 5, 10, 11, 14, 16, 17, h^2^>78%, Table 1), all of them being binomial characters. But even for these characters, some within-strain variation could be observed with only few exceptions such as for instance the alignment of a guard setae on PAO for SH, BR, GB, TO and US (N°1), the presence of additional sensilla on Ant. 3 (n° 5) for SH, AP, BR, BV, GB, WI, the presence of a clawtooth (n° 10) for SH, BR, DK or the number of ventral setae on thorax 3 for strains SH and DK (n° 11). Because of this within-strain variation, the assignment of a unique and clear-cut value for each clone-character pair is not straightforward.

### Phylogenetic and distance trees

The parsimony analysis yielded two primary trees. We knew from a previous study that the eleven clonal strains group themselves into two distinct clades [9]. After artificially rooting our morphologically based phylogenetic tree, we found that our strains organised themselves again into two clades that correspond to the previously described clades: AP, GB, HA, BR and BV in clade "A" and DK, GM, PB, TO, US & WI for clade "B" (Fig 5A). The strict consensus tree has two multifurcations on clade B. In this analysis the sexual strain SH branches itself close to the clone GB. Based on morphology, the clone AP branches at the base of clade A. This was also the case in our previous molecular phylogeny [9].

The overall topology of our cluster dendrogram (Fig 5B) was found to be very similar to the phylogenetic cladogram: the two lineages (A & B) are clearly supported. And even within these two lineages, some similarity can be observed: the similarity between clones PB and GM or between BR and GB is supported for instance by both approaches (Fig 5). The main difference between the two topologies is the position of the sexual strain SH which roots itself outside the two clades in the dendrogram whereas it is closely linked to clone GB in the cladogram. This discrepancy is probably due to the character n°12 which provides no information in the cladistic analysis whereas it moves SH away from the other strains on the distance tree (Fig 5C).

## Discussion

### Taxonomical status of strains

All the strains under study fit quite well the modern taxonomical understanding of the species *F*. *candida* sensu strictu [50]. All strains have characteristic long furca, the same sensillar chaetotaxy, ventral setae on third thoracic segment, 13–29 setae on anterior side of manubrium and other characters that define well this species (for more details see the redescriptions mentioned above and [50]. In our individuals, the following characters differ a little from that is written in the diagnosis compiled by Potapov for this species in more traditional and wider understanding [27]: number of apical setae on posterior part of manubrium (1–5 versus 2), number of anterior setae on manubrium (11–27 versus 16–32), sensilla on first antennal segment (4–5 versus 3), ventral setae on third thoracic segment (1–4+1–4 versus 2–3+2–3). No doubt, our strains don’t represent all possible variability of this species sensu lato.

### Variability and heritabilities of the characters

All characters vary strongly depending on individuals. This yields to considerable overlapping between clones in almost all characters. But most of the characters studied here displayed some level of genetic variability. The range of within-strain variation of the four characters with no genetic variation was quite high and this may explain the absence of any significant heritability. The heritabilities of the other characters varied from comparatively low values to very high values (from ~7% up to 94%). It is interesting to note that the heritabilities never reached 100%, which means that all of the heritable morphological characters that we have studied were not absolutely stable for every lineage. To our knowledge these are the first measurements of morphological characters’ heritabilities in a Collembola.

The large range of measured heritabilities can reflect the fact that the evolution and divergence of morphological characters among isolated clonal strains happens at different rates: some characters have already diverged a lot while for others the emergence of genetic variability may still be at its beginning if it is ever going to evolve. But it can also result from different levels of phenotypic canalisation among the characters. For the same level of genetic variability, a high canalisation is associated with a higher heritability due to a lower level of within-lineage character variation. Overall, this character diversification reflects the polychaetotic or oligochaetotic evolutionary tendencies within this group of *F*. *candida* strains (apart from characters n°1 and 10).

### Strain identification by morphology

According to our analysis, between 9 and 13 of 18 morphological characters can be used to discriminate the strains depending on the pair of strains one wants to compare. These characters may be strongly age-dependent and should only be used among the individuals of the same age or instar [57]. Most of them are a number of setae on different parts of the body.

These morphological characters could be profitably used to verify that two or more strains belong to the same clade or to quantify their morphological difference. This could be useful, for instance, for assessing the validity of comparison of multiple ecotoxicological tests performed on different strains. It could complement the use of molecular tools for genetic characterisation of the strains [41]. These morphological characters could also be used if one wants to run laboratory experiments where several strains are mixed together in competition experiments for example [58]. Using an appropriate character and pair of clones, it is possible to identify a strain using a single individual. For instance, one individual of the strains BR, BV and GB can be clearly distinguished from an individual of strain US by counting fewer or more than 24 setae on Ant.I (n° 3) respectively.

But for most comparisons, drawing a robust conclusion means studying more than one individual. To distinguish two strains the choice of characters will depend on how many individuals can be measured. If one wants to identify individuals belonging to several strains mixed together in a single population, one will either need to use very discriminant characters or to breed the isolated individuals to make more measurements on less discriminant characters on their numerous descendants.

### Patterns of morphological and molecular evolution

Using analysis based on the genetically variable morphological characters, we found that the variability of morphological characters carries some phylogenetic signal and can be used to study the patterns of evolution of the different lineages within a single species. The two methods we used to build-up the trees (cladistic and distance) produced very similar results.

The studied strains first split into two distinct clades and then diverged within each clade. We have again found the two major clades that have been previously found using molecular phylogeny [9].

The congruence between the topology of the distance tree and the cladistic tree is striking. The topology is similar at a broad level (the two main clades) but also at a closer look. In our previous work using some genetic markers [9], the genetic signal was insufficient to provide us with some details regarding the fine topology of the tree within each clade. More work needs to be done using molecular analysis to tell whether the congruence between the tree based on molecular and morphological data persists at a thinner phylogenetic scale. But we can conclude from this work that morphological characters, even if they display a lot of variation within a population or within a strain, can provide in this species reliable and meaningful information regarding the identification of clonal strains and the reconstruction of their evolutionary relationships.

### The evolution of setosity

If we compare on average the evolutionary tendency of the chaetotaxy of the strains between the two clades, it appears that clade A tends to be oligochaetotic while the second clade tends to be more polychaetotic. This pattern is striking for characters n° 2, 3, 5, 16, 18 while the reverse tendency only appears for character n°11. The lineages with a slow life history (A) are less hairy than the fast ones (B). Is this morphological evolution adaptive and if yes what is the adaptive significance of this pattern? The two clades have very contrasted life-history strategies and are thus believed to have adapted to contrasted environments [45,59].

During this evolutionary divergence, it is possible that multiple small-effect mutations controlling the level of setosity on different parts on the body have arisen independently and have been selected differently within the two major clades. Under this adaptive scenario, the level of setosity could be considered as an adaptation, and selection should also act to reduce the developmental variance by favouring robust genotypes producing more canalised phenotypes whose level of setosity is adapted to a specific environment [60]. But from this viewpoint, the adaptive significance of this morphological evolution remains to be determined. However the observed pattern of phenotypic variation could also result from the fixation of a single or few mutations of relatively large effects that control the level of setosity globally, or at least on several parts of the body as has been shown to be the case for *Drosophila* [61,62].

In such a case, the observed difference in the mean level of setosity between the two clades would not necessarily be adaptive since it could simply result from the fixation by chance of one of few alleles controlling the number of setae on different parts of the body in the common ancestor of one of the two clades.

Lastly one can question the genetic basis on which the evolution of setosity in *Folsomia candida* depends. Much work has been done on the genetic mechanisms underlying bristle patterns in Diptera and especially in *Drosophila* [63,64]. The *achaete-scute* (ac-sc) gene codes for a transcription factor which plays a central role in determining the development of the fly's macrochaetes [63]. The position of bristles on the thorax is determined by the spatial expression of this *achaete-scute* gene and this expression is itself controlled by many independent cis-regulatroy elements [64–66]. Mutations in these cis-regulatory regions can affect some of the thoracic bristles in *Drosophila* [61,66,67] and the variation of the number and arrangement of these bristles in different species of Diptera seems to be driven by the evolution of these cis-regulatory sequences [63,64].

One can make use of these results when studying the genetic basis of the evolution of setosity in *Folsomia candida*. Genomic tools developed for springtail*s* and the recently sequenced and analysed transcriptome of *F*. *candida* [68] could be used in order to search for an *ac-sc* homologue and its associated cis-regulatory regions which must exist in *Folsomia* genome given that both the structure and function of the *ac-sc* genes are known to be conserved in many animal species [63,64,69]. This could then be used for studying whether the observed phenotypic divergence in the setosity rely, as for *Drosophila*, on mutations in cis-regulatory elements regulating the transcription of a collembolan homologous of the *ac-sc* gene [64].

More work is required (i) to study the selective advantages that could be provided by various levels of setosity under different environmental conditions, (ii) to study the adaptive significance of the observed morphological development noise and (iii) to search for the genetic determinants underlying these phenotypic variations and assess whether these morphological changes are driven by the evolution of cis-regulation.

### A speciation in progress?

We knew that *F*. *candida* morphospecies comprises a great number of life-history diverging evolutionary lineages [45,59,70]. We observed some phenotypic variation on many morphological traits and found that the lineages also diverged morphologically. This is a good evidence that morphological speciation is in progress.

We observed that the evolution of new phenotypes in diverging lineages of *F*. *candida* is accompanied by a high developmental noise (non genetic phenotypic variability) since for each lineage, the diverging morphological traits are still unstable and are partly overlapping. How this variability may evolve during what could be an on-going speciation remains to be addressed. Moreover, this divergent process may be blurred and slowed down by genetic transfers between the diverging lineages driven by the sexual strains if they can intercross with some of these lineages. But the precise position and role of these sexual strains in the evolutionary history of this species remains at present unsettled.

### Cryptic diversity and potentialities of morphology in Collembola

A high level of cryptic diversity within already known Collembola is often postulated in the current publications [7,8,71,72]. At whole for Collembola, the unexpected genetic divergence was revealed within homogeneous morphospecies. As a result, several common and widely distributed species passed to the “complexes” of many potential species: *Parisotoma notabilis*, *Friesea grisea*, *Heteromurus major*, *Ceratophysella denticulata* and others. It was concluded that modern morphological taxonomy underestimates the number of species. We showed in our study that the morphological characters could be successfully found and used to identify even the intraspecific forms, which are the strains in our study. The considerable overlapping between clones in almost all characters makes the identification of clones less exact but possible. With this aim in mind we must render the morphological noise as small as possible, particularly by culturing the individuals in the same conditions and by examining individuals of the same age, that can partly be applied to the species mentioned above.

## Supporting Information

S1 Dataset (ZIP)
